# Leadership Development Through Exploring Critical Perspectives and Storytelling in Pop Culture: Toward Leadership for Liberation Values

**DOI:** 10.1002/yd.20662

**Published:** 2025-02-25

**Authors:** Kathleen Callahan, Sean Connable

**Affiliations:** ^1^ Department of Leadership and American Studies Christopher Newport University Newport News Virginia USA; ^2^ Department of Communication Christopher Newport University Newport News Virginia USA

## Abstract

Popular culture exists as an expression of cultural history. It speaks to who we are, what we aspire toward, and where our generation stands in relation to the major issues of the day. This article is a conversation about the myriad perspectives offered in this issue of *New Directions for Student Leadership*, exploring the contributions each makes to the study of leadership and leadership development, engages with popular culture as an important tool in leadership education and development, and explores some of the limitations that have risen with using popular culture, regardless of the form used. Furthermore, the conversation will explore how a leadership pedagogy, rooted in popular culture, has the potential to serve as a transgressional space, creating a place where the voices of the marginalized and minoritized might be heard and better understood.

## 1 Introduction

Popular culture exists as the expression of who we are, what we aspire toward, and where a generation stands in relation to the major issues of the day. In many ways, it exists as a living laboratory, a place where we can come together and explore, question, dream, and learn. This issue of *New Directions in Student Leadership (NDSL)* is a result of a love of pop culture and leadership educators and practitioners wanting direction in how to engage with different forms. The articles offered in this issue exist within a series of interesting tensions between who we are (the stories we tell, be it via lyrics, a podcast, a comic book, a screen, or a table with a handful of dice) and where these stories seem to lead us time and again—to a place where we seek to better understand who we are, individually and together, characters in the same story.

This issue, gentle reader, is a product of many conversations between classes or over a latte between Kat and Sean. Every fall, we return from a summer of leisure (insert academic sarcasm), and our first conversations often stray toward the last Marvel movie we watched, or the latest Star Wars series on Disney+ and the opportunities we see in those stories to do something exciting in the classroom and with students. We have long talked about how to team teach a course that looks at popular culture, persuasion, and leadership grounded in a list of pop culture artifacts that seem to be growing. In that spirit, we would like to approach this article as a conversation of what we have learned, and we encourage anyone reading this article to engage in the issue as a whole and as individual approaches or ways to engage in pop culture for leadership development. Before we begin our conversation, we feel it is only fair that we introduce ourselves and our positionality coming into this space.

## 2 A Conversation

*Kat*: It's me. Hi. I'm the problem, it's me. I took on the role of Editor because, simply put, I am a complete pop culture nerd. I love music, movies, television, sports, and social media, and I absolutely love to teach leadership through pop culture examples like *Star Wars, Black Panther*, and *Barbie*, not to mention my semesterly Spotify playlist that all students contribute to so we can diversify our listening. I approached this issue with a few things in mind: (1) interdisciplinary lens, (2) critical approaches to leadership, (3) a wide net of popular culture, (4) leadership development pedagogy for in and outside of a classroom, and (5) awesome colleagues who are passionate about their article topic. I sought out a Co‐Editor that would balance me out in a few ways. One, he adds depth of pop culture nerdiness with his love of Comics, D&D, and gaming, and two, he is a Rhetorician in the Department of Communication who used to teach Leadership Studies, adding an interdisciplinary lens.

I grew up on a college campus where my mother worked in student affairs, and in a high school where my father coached basketball and taught history, in my hometown in NC, a place that made me the person that loves music, sports, history, and education. My educational background is in student affairs and higher education which led me to the role of leadership educator. As a cisgender white woman, I am acutely aware of the privileges I hold, and I intentionally strive to ensure that my teaching materials reflect diverse identities and experiences, rather than reinforcing those aligned with my own. All of these things make me Kat, and, as an educator, I want to incorporate examples that are not only relevant to my students but also capable of challenging our collective perspectives and pushing beyond established comfort zones.

*Sean*: Hello! I'm the problem child. To be honest, I'm the old man of this duo. I stepped onto this project because, as alluded to above, Kat is one of my dearest nerdy friends. If you want a really clear picture of the culture that shaped my world, just look at the kids from Stranger Things and strip out the monsters and the government conspiracy. I grew up as a cisgender white male in the South during the satanic panic and was shaped by the misfits of the Breakfast Club and Luke Skywalker, the erstwhile Jedi. I have not taught leadership in nearly a decade, as most of my work is more directly related to rhetoric and the study of persuasion. At the same time, popular culture is an ever‐present part of what I do. In the classroom, I teach how comic books are used as social critique, helping students connect persuasion to cultural narratives that make up the world in which they reside.

At home, I have three kids that challenge me to explore our collective story, as it's being written. I talk with them about friendship as we read through Harry Potter, we journey with the fellowship in search of the one ring, and they've helped me learn to believe in heroes again as we watch and cheer as the Avengers assemble. One thing that I keep circling back to over the years, is that while popular culture seems to be ever evolving, many of the stories remain the same. *The Goonies* became *the Sandlot*, became surviving adolescence in *We Are Lady Parts*, and then became the adventuring party on *Stranger Things* or friends overcoming tragedy in *Reservation Dogs*. Many of the stories we share are woven with the ‘coming of age’ threads.

### 2.1 Beginnings and Perspectives

*Kat*: So where do we go from here, Sean?

*Sean*: Maybe we should start at the beginning.

*Kat* (*starts singing like Julie Andrews)*: Let's start from the very beginning, a very good place to start. This may be one example of a pop culture reference that may fall flat to some.

*Sean*: I think the thing that struck me most about this project was the multiplicity of perspectives. Yes, there was a common thread… popular culture… but how it was incorporated, and, more importantly, some of the avenues of inquiry it introduced or opened up were unexpected. For those of you who have made it all the way to the end, I think how we structured this journal speaks to the rich diversity of ways that popular culture can be incorporated into the study of leadership. In some cases, we see it functioning as a tool. It's the instrument or the object of study. It's the case we bring into the classroom to make sense of the concept or practice… it's the thing we observe. In another real sense, pop culture points to who we *are*, it's the expression of our cultural history or the shared story that defines us. Schmidt et al. (2025) do a solid job here setting this up. In their article on using films, they lay out clear lines between the films we watch and the leadership competencies that we can draw from them. But again, thinking about that multiplicity of perspectives, how film can be used as more than just a tool. It is a source of cultural representation, and it also reflects the stories we use as a culture to construct the meaning of these incredibly nuanced concepts and ideas. Some of our other authors, like Raffi Sarkissian and you, Kat, explore some of these other perspectives as well, providing a rich pedagogical framework from which work.

*Kat*: Regardless of the form that pop culture takes, we can see how it can be used to connect with students. We can use specific tools like TikTok. I am not that comfortable with it beyond seeing them a couple weeks later on Instagram Reels, but Jennifer Billinson (2025) is an expert. She gives us some insight of the many ways we can look to TikTok as a place where leadership happens—like how politicians interact with followers, and how we can use it to develop leadership skills—like how we can use videos to teach others. Podcasts are another great tool. It feels like we are doing that now. But to have some of the leading podcast voices write this article feels pretty awesome.

Devies et al. (2025) are a gold‐winning team of leadership podcasters and podcast editors, and they not only give us insights into using podcasts as an instructional tool in leadership development, but they also connect instructional and assessment strategies with podcasts and components of the leadership learning framework. This makes it easy for educators and practitioners to look at their learning outcomes within the leadership learning framework and use what they are already doing, like case studies, group projects, interviews, reflective journaling, and so forth, and then use podcasts to elevate learning. I do this in my Leadership Through the Ages course, and students love to create podcasts about historical figures, and they get very creative. I love history, but having these students create podcasts with their classmates and pretend that they are interviewing Ching Shih, a powerful pirate, just makes it fun and something they will not forget. Engaging in creative outlets is one way that students won't forget what they are learning, which makes me think of D&D.

### 2.2 The Creation of Shared Stories

*Sean*: It's interesting. I've never thought about D&D as a pedagogical tool, per se. What's fascinating about what you just mentioned, however, is that TikTok and podcasts both share something in common with Dungeons and Dragons when we consider how they might be used in a classroom setting. All of them are an act of creation. I think Lasley et al. (2025) do a good job explaining the pedagogical mechanics and possibilities behind D&D, but what I think really makes it a potentially effective tool in the study of leadership is how it works. Dungeons and Dragons is *collaborative* storytelling. Rather than being something that we watch or read… it's something that we create together. The players and the DM sit around a table and tell a story that can put leadership into *praxis*. Let me see if I can explain this. In one of the games I ran with some of my colleagues (yes, friends, a table full of professors—but not Kat, she doesn't stay up that late), they were charged with a heist of sorts.

They come up with a plan on how they want to pull off said heist, but the plan is subject to randomness and probability. When the dice are rolled, the thing they planned fails… spectacularly. What results over the next few minutes is leadership in practice. They adjust on the fly and solve a now actively evolving problem. They make ethical decisions and then sit with the potential consequences. They do this, not as themselves, but as characters with stories and motivations all their own. It creates a space that's safe to try and fail… a space that allows people to test decisions that they might not have made if the stakes were real.

Moreover, the experience can be a transformative one! The authors emphasize the potential for how roleplaying games like D&D create the space to transform the player. They can have a therapeutic effect, allowing players to deal with difficult issues or subjects of deep concern by approaching them from behind the facade of a character. To be honest, it's something I want to try… but I would have to figure out some ways to work with the limitations of the game system. At the same time, you can tell whatever story you like, so it makes space to explore difficult subjects or problematic issues like power and ethics. It has tremendous potential.

### 2.3 Interdisciplinarity

*Kat*: One of my favorite things about working on this issue is simply tapping into different disciplines which include the authors but also their passions. We know leadership is interdisciplinary, but leadership exists in every aspect of our lives. Knowing the background of the next three authors, Cruz, Vermont, and Watkins (Cruz et al. 2025), they all have a love for visual art, and I think for each of them, that passion has landed them into a research area that is still new and growing within the leadership scholarship.

Not only do they dive into art activism as leadership development, but they discuss the student leader activist identity continuum (SLAIC; Bruce et al. 2019). We see the importance of identity development in leadership in this issue and we have multiple authors dive deeply into the leadership identity development model (Komives et al. 2005), but the SLAIC is a new approach that can directly impact our students. One of the fascinating aspects of art is simply the fact that it isn't just about the artist's work but also about how they involve their identity in their work.

*Sean*: After all, art is both a tool and a story, isn't it? It's an object that has tangible effect, but at the same time, is a product of *who* the artist is. Their identity is as much a part of the art as the message they create.

*Kat*: For my students who identify with or see themselves as artists or possible artists, it is incredibly powerful to observe them see themselves as transformative leaders that, “…interrogate systems and structures… while centering justice and equity,” (Cruz et al. 2025, 8). The same systems that artists critique and interrogate are a thread that runs through popular culture and why sometimes we, the audience, feel so connected with people's stories. We can see ourselves being held back by similar systems, and we feel connected to those who are doing their best to persist, resist, and move forward. And, sometimes, we acknowledge the artists’ humanity and need for rest or connection that draws on their authenticity. Once again, the human story is relatable, and pop culture gives us examples in every form. This is a great transition to begin to talk about a major theme we noticed in almost all the articles: pop culture used as storytelling.

It helps us as individuals analyze our own identities and how we are represented in the world (or on‐screen). We saw the use of leadership models including the leadership identity development model, culturally relevant leadership learning model, leadership for liberation, and the use of critical perspectives in how we make meaning of certain forms of pop culture. In Patterson‐Stephens, Beatty, and Smith's (Patterson‐Stephens et al. 2025) article *Creating to Transgress*, which we will discuss shortly, they call attention to Harper and Kezar's (2021) *Leadership for Liberation*. This framework focuses on the need for transformative leadership in higher education, particularly centered on equity, inclusion, and social justice (Harper and Kezar 2021).

It emphasizes how leadership can actively challenge and dismantle oppressive systems, while aiming for the liberation of marginalized and underrepresented communities, and they include six liberatory values: “…liberation, power and oppression acknowledgment, system challenging, storytelling, support networks, and fellowship,” (Harper and Kezar 2021, 2). Every form of pop culture can address these values, and I hope you are able to see how each is addressed in different ways through articles in this issue. But now, we will turn our attention to storytelling and representation.

### 2.4 Representation

*Sean*: To start, in some ways, I think we need to address the role that representation plays, particularly insofar as our storytelling is concerned. Sarkissian's background is in critical media studies, and what he brings to the table is a real understanding of what organic representation is, but he also speaks to how marginalized communities are stakeholders in the creation of pop culture narratives. In particular, I think he really puts his finger on how underrepresented populations, in the act of telling their story, serve to change the way we think about production, traditional models of how they see and understand the underrepresented group by creating a space where we can discourse *with* the groups in question. When we think about this in terms of Harper and Kezar's (2021) work *Leadership for Liberation*, it seems a natural fit. Where critical media studies seek to understand the intersection of industry, production, and representation,

Sarkissian (2025) shows us how the creation of these stories can subsequently be an act of leadership, and how using a more critical lens to think about storytelling in this way can help us to think about popular culture as *more* than just a tool. It becomes an act of leadership—a pedagogy that helps us to better understand what producing these types of narratives requires. A co‐creation with the other that respects and speaks to marginalized perspectives, but one that breaks traditional models and instead is presented, “…with and for the communities they represent,” (Sarkissian 2025, 12).

*Kat*: Originally, Sarkissian and I were working on an article together when we realized the depth of the topics we wanted to cover, we decided two articles were more appropriate. When thinking about an article I wanted to contribute, I thought about my women in leadership course. Students enjoy the course because it is, for some, the first time, they have the ability to study women in leadership as the central theme. It is shocking how little representation women receive in classrooms and when discussing leadership broadly.

My question became: How do we see, explain, and analyze leadership in female protagonists? Hinck et al. (2023) found, “…stories (1) allowed deeper cognitive learning, (2) provided models of authenticity and vulnerability that encouraged vulnerability mirroring, (3) helped instructors regain recoverable loss through the telling of their stories, and (4) anchored learning in the affective zone for students upon which further learning and group cohesion occurred” (para 1). We know that stories allow for deeper leadership learning and the authors in this issue have given us some guidance for how to be inclusive of stories in pedagogy. With that in mind, I was set on providing examples of women, which is not so difficult if you are intentional. All around the same time, films like *Black Panther: Wakanda Forever* and *Everything Everywhere All at Once and* shows like *Ms. Marvel* and *We Are Lady Parts* captured my attention, I was excited to see complex and intersectional women as protagonists and their stories front and center.

Beyonce and Taylor Swift sold out arenas while Greta Gerwig and Barbie hit me in the feels reminding me of so many of my own insecurities but also my ability to stand tall. Most recently, women in sports have dominated the media, including the women of the 2024 Olympics, to college volleyball and basketball, selling out arenas to the WNBA and women's tennis stars getting their due recognition, and finally, the US Women's Soccer team achieving pay equity. Stories of Simone Biles, Ilona Maher, Katie Ledecky, and Sha'Carri Richardson winning medals and hearts gave me so much energy to highlight women and their complex stories.

In *Femininomenon* (thanks, Chappell Roan), I discuss the desire to see more complex and intersectional storylines for women through examples of *Barbie* and *Ashoka*. Each of these examples is focused on the main character but also looks at the related ‘universe’ involved and who is writing, directing, and producing these titles. Just as I looked at Barbie and Ashoka as individuals, I think it might be relevant to talk about the individual icons. How do we look at celebrities, sports stars, musicians, and other influential people? Let's start with celebrities themselves as an overarching storyline.

### 2.5 Celebrity

*Sean*: I always find the subject of celebrity complicated at best and problematic at worst. First, we to see the celebrity as both a creator and product of popular culture. Secondly, as Devies et al. (2025) suggested, celebrities, like any human subject, are inherently flawed. While this might provide some challenge depending on the nature of the flaws, it also offers an opportunity to explore leadership through the lens of success and failure and the “complexities of their humanhood and identity as leaders” (Devies et al. 2025, 2).

A perspective that I found particularly salient (not to mention one that intersects with some of my own work), is that of the celebrity as a product of popular culture. They both constitute pop culture but also reflect the social construction of that culture as well. This provides fertile ground to think about how the celebrity exemplifies leadership as a reflection of the culture, or, in some cases, demonstrates leadership by changing the culture in new ways.

Oftentimes, the demonstrated acts of leadership provide inroads to more carefully and critically think about leadership in culturally relevant ways. Depending on how the celebrity demonstrates leadership, their culturally relevant platform provides a means of adding to the ever‐growing and involving social narrative of leadership. That, I think, is one of the real contributions offered by Dr. Devies and her colleagues. One might even argue that our students’ article on Beyonce and Taylor Swift touches on some of these very notions.

*Kat*: Tapping into specific examples of celebrities, Tucker and Vincent (2025) bring the musicians Beyonce and Taylor Swift to the center of conversation (where they usually are in the media as well). Love them or not, these two have stories to tell through their musical journeys, lyrical messages, and in their interactions with fans, the public, and haters. Leadership concepts can be analyzed using them as case studies, and the authors highlight how music can connect individuals in many ways.

Shollen and Hanold (2025) did something similar in their article exploring the impact of sports on leadership development and offering particular athletes as case studies to be analyzed. They use famous names in sports like Messi, Caitlyn Clark, and Venus Williams, and they highlight Togethrx, one of my favorite brands, started by famous athletes Sue Bird, Simone Manuel, Chloe Kim, and Alex Morgan to address equality, diversity, and impact in women's sports. Case studies can be a way to have learners analyze leadership concepts in meaningful ways and connect them to their favorite icons. But one of the big limitations when it comes to all things pop culture is that not everyone connects with things like music or sports. Sean, take us to another form of pop culture.

### 2.6 Narrative

*Sean*: I think this brings us to my piece. In particular, I wanted to offer something that my own field might contribute to this conversation. While I think we often talk about the power of stories, we tend to think about stories as an object, or currency, used to acquire social capital. Think about how many presidential candidates, for instance, use some derivation of the American myth when they run for office—it has a lot of power, right? One little tool that helps us to see commonality in places where it might not have first existed. It helps to frame or stage the politicians’ policies in such a way that they seem a natural outgrowth of the story in question.

But if you really think about it, the stories we tell also serve a larger persuasive function. All the way back in 1984, Walter Fisher contended that our stories also served, collectively, to function as a mode of rational decision‐making. Not traditional rationality, bound up in a set of logical arguments, mind you, but rather what he called a logic of good reasons. Think about it this way. How do most of us define what a hero is? It seems simple enough, right? If you think carefully about the question, you might realize that most of us don't have a set of traditional arguments to define something as heroic.

What we have, in truth, is a set of stories that we've told, as a culture, about heroism. Those stories then serve as a lens through which we see and come to symbolically construct heroism. Some do something and we ask ourselves, “is that consistent with stories I've heard about heroism?” Or, if someone is defined as a hero, then we can use the same stories to predict the kinds of behaviors they will engage in, as they *should* be consistent with other stories of heroic action. Fisher called this framework the narrative paradigm.

So, what does this have to do with leadership and popular culture? Well, when we think about leadership, what is it really? Are there a set of concrete arguments to define what a leader is? I would contend that leadership, in many ways, is a logic of good reasons.

If we think about leadership as narratively constructed, then it's simply a matter of locating stories that speak to and construct our idea of leadership. For me, comics have always been one of those stories. What's exciting for me as an educator is that Fisher's narrative paradigm asks us to investigate these stories differently. The comic book finds utility in that it helps us to better understand who *we* are by the way we collectively understand leadership and how that understanding has changed across time. The best part is that this can be connected to *any* story as a part of a larger set of cultural narratives. We can look at how comic books shape the heroic, how we tell the story of the leader, or how other forms of storytelling, like hip‐hop, tell us about who the culture out of which those narratives rise.

*Kat*: Starting on August 11, 2023, hip hop celebrated its 50th anniversary in a year‐long celebration of cultural events, concerts, and conversation. Even as a young form of art, hip hop has created so much to culture. Ford et al. (2025) discuss the five elements of hip hop culture and connect them to the Black student experience, “…demonstrating how it functions as a cultural and intellectual venue for self‐expression, empowerment, and leadership development,” (1). They use storytelling and their own experiences to connect hip hop culture, identity, and how it influences their role as leadership educators and then expand on how to use hip hop as a tool for leadership development (Ford et al. 2025).

Culturally relevant leadership learning and critical pedagogy emphasize the need to center lived experiences and identities of marginalized communities, and hip hop provides a platform to explore through music, art, and expression (Akom 2009; Bertrand Jones et al. 2016; Freire 1970). Hip hop's emphasis on authenticity, resilience, and self‐expression nurtures leaders and followers who are not only culturally aware but also committed to social justice. Using hip hop as pedagogy is another way to bring in popular culture to cultivate leadership development that is culturally relevant, critically engaged, and geared toward transformation. Hip hop is both a tool and a social movement.

### 2.7 Transformation

*Sean*: Story as tool, and then story as lens… it seems only appropriate that we end this conversation by looking at story as a mode of transformation. Patterson‐Stephens et al. (2025) offered us a vision of what leadership as a culturally aware transformative mode of praxis looks like. For me, it seems the truest picture of what many of us are attempting to arrive at in the classroom, be it teaching leadership, communication, or any other subject that is mindful of the larger world we're a part of. It speaks to a mode of teaching bound in letting the voices and stories of others be heard.

The authors reminded us, like Sarkissian (2025), that representation matters, and, at times, the academy itself is what creates issues or fails to adequately address the issue of underrepresentation. In the one space where we should see space being made for students and scholars to find their voice, they are often restricted, if not silenced. They also make a strong argument for leadership for liberation through media praxis and how it not only creates space for careful, critical thought, but also how creating those spaces allows students and advocates to find agency—to find their voice and create a space where identification with others is possible. The act of media creation, as an act of liberation, becomes a laboratory where students can learn to not only think about these issues, but actively work to challenge them. At times, as a scholar I struggle to see how we might put theory to praxis—Patterson‐Stephens et al. (2025) do just that, using popular culture as the vehicle for education. What do you think, Kat?

*Kat*: I think we have found a very effective pedagogical vehicle to use for leadership development. Using pop culture as the vehicle, two central themes that have come out of almost every article are of storytelling and the need for critical perspectives in leadership development.

Applying leadership for liberation, educators and practitioners have the opportunity to engage in the liberation values and concepts of liberation, power and oppression acknowledgment, system challenging, and storytelling in all forms of pop culture (Harper and Kezar 2021). Celebrities, musicians, and sports figures frequently embody and engage with the values central to leadership for liberation, often using their platforms to promote social justice, equity, and empowerment (Harper and Kezar 2021). Digital tools and platforms such as TikTok, podcasts, and creative outlets like Dungeons & Dragons (D&D) offer participatory spaces for individuals to explore and engage in critical dialogues about liberation, identity, and power dynamics.

Moreover, films and television serve as powerful cultural artifacts, providing practical illustrations of leadership for liberation, offering narratives and examples that reflect and challenge societal structures and inspire transformative action. What has been created through engagement with each of these mediums are a result of or can create the final two liberation values and concepts: support networks and fellowship (Harper and Kezar 2021). These support networks, whether in communities of fans, social media interactions, or through leadership development, create spaces where shared stories and mutual support foster resilience. The fellowship that arises in these spaces not only encourages learners to engage in personal transformation but also to contribute to the collective empowerment of their community. But we should address some collective critiques and limitations.

## 3 Critiques and Limitations

*Sean*: In many ways, the challenge of using popular culture is rooted in its ephemeral nature. While it could be argued that the narratives or the stories themselves are long reaching cultural expressions, *how* those narratives are portrayed and how they are evolving with time play out in their depiction within popular culture. Remember, cultural history is the story of the generation telling it. Think about some of these stories we once told, or the way they told them, and how poorly they translate to today. Dr. Seuss, for instance, while having written delightful stories for children also, at times, demonstrates the culturally problematic ways his generation looked at race in some of his writing and illustrations. One way to address this issue is to own how the culture has changed, and how it changes the way we see and understand popular cultural references. It may require you (namely me… the old one in the room), to adopt more currently relevant examples, as popular culture has a shelf life, or shift how you address dated references as examples of perspectives that have themselves shifted or changed.

This leads to the second major limitation, namely that popular culture needs to be staged or framed when used as a pedagogical tool. In my own chapter, when we think about how comic books are used as a way of talking about leadership, the story itself needs to be framed. There is a lot of work that needs to be done on the front end, setting up the story and characters for the students to understand, as well as on the back end, which includes adequate debriefing to ensure that students are drawing the relevant lessons from the text. Given that much of popular culture is symbolic, there is no shortage of interpretations that can be made, so the role of the educator or facilitator is to focus interpretations of the relevant ideas, challenges, or questions. This also provides an excellent opportunity for us to make space for those in the classroom, making sure that we address issues of representation, power, as well as the voices of the marginalized and minoritized to be heard. In terms of the how, Jesse Ford and his co‐authors (Ford et al. 2025) offer an easy way to see how this might be done, by connecting relevant learning objectives, perspectives, or theories to a hip‐hop text, then drawing on students’ experiences and interpretations of the text. Think of it less as the application of a tool, per se, and more the adoption of a perspective, one where hip‐hop becomes both the interpretive lens, as well as the text being analyzed.

*Kat*: Additional limitations include both access, interest, and point of view or positionality of those involved. Not all learners have access to the tools, films, television shows, podcasts, games, and other digital media referenced in this issue, as many forms of popular culture remain restricted behind paywalls, limiting availability. In this issue, different articles note different resources that can get around some of these limitations, like Lasely et al. (2025), who note free access to an introductory story by Dungeons and Dragons as well as utilizing your institutional resources through Libraries that have access to a wide variety of digital resources.

Some learners may not demonstrate an interest in certain forms of popular culture introduced in educational settings, which can hinder their engagement with leadership concepts. These barriers highlight the need for educators to consider accessibility and diverse interests when incorporating popular culture into pedagogical practices. This places the responsibility on educators and practitioners to actively seek out diverse and accessible examples within popular culture. It also requires them to stay informed about emerging trends and evolving forms of media. While it may be convenient to rely on examples that have been effective over the past 20 years, educators must ask whether doing so truly serves the best interests of student learning and leadership development. Staying current and incorporating relevant, contemporary pop culture not only enhances engagement but also ensures that educational content resonates with students' lived experiences and evolving cultural contexts. One way to do this is accessing and engaging with podcasts mentioned in *Sound Leadership* that can keep you abreast of new and emerging trends in leadership as well as pedagogical tools, people, and ideas (Devies et al. 2025) or simply by engaging with social media intentionally (aka get outside of your algorithm of dog videos, recipes, and CrossFit workouts, I know it isn't just me).

Finally, a significant limitation of this final work is the positionality of the authors as cisgender, heterosexual, white scholars. This also applies to who is involved in creating the different pop culture mediums, for example, how men may write a female character for a comic or film, as Connable (2025) comments on with the creation of Wonder Woman in his article. As highlighted by Patterson‐Stephens et al. (2025), students, faculty, and staff of color navigate and experience higher education in ways that differ from those of their white counterparts. Consequently, our personal perspectives and reflections may unintentionally overlook or inadequately address certain concepts, topics, or experiences relevant to individuals from racially and culturally diverse backgrounds. Critical conversations and collaboration in an intentionally interdisciplinary and intersectional way serve everyone. These approaches ensure that diverse perspectives and experiences are acknowledged and valued, enriching understanding and fostering more inclusive learning for all involved. Practically, this can be done by determining your own positionality while taking stock of those involved in the process (writers, directors, creators, etc.) to fully analyze media critically to discern underlying messages about power, responsibility, or social identities. This critical media literacy can help to foster thoughtful and reflective leaders.

## 4 Conclusion

We encourage educators and scholars to build upon the insights presented in this issue of the NDSL by further exploring and addressing the limitations discussed. We hope this work serves as a foundation for continued inquiry into more inclusive and equitable practices in leadership development. Pop culture offers significant pedagogical value, presenting numerous opportunities for educators and practitioners to engage with students and leadership learners in meaningful ways. By incorporating different forms of popular culture into the pedagogy, educators, and practitioners can foster stronger connections with students, leveraging these shared cultural references to enhance engagement and deepen their understanding of leadership concepts. Utilizing pop culture as a tool and as a lens not only makes learning more relatable but also allows educators and practitioners to capitalize on its relevance to contemporary societal issues and identity formation.
